# Red Sea Coral Reef Trajectories over 2 Decades Suggest Increasing Community Homogenization and Decline in Coral Size

**DOI:** 10.1371/journal.pone.0038396

**Published:** 2012-05-31

**Authors:** Bernhard M. Riegl, Andrew W. Bruckner, Gwilym P. Rowlands, Sam J. Purkis, Philip Renaud

**Affiliations:** 1 National Coral Reef Institute, Nova Southeastern University, Dania Beach, Florida, United States of America; 2 Khaled bin Sultan Living Oceans Foundation, Landover, Maryland, United States of America; Heriot-Watt University, United Kingdom

## Abstract

Three independent line intercept transect surveys on northern Red Sea reef slopes conducted in 1988/9 and 1997/8 in Egypt and from 2006–9 in Saudi Arabia were used to compare community patterns and coral size. Coral communities showed scale-dependent variability, highest at fine spatial and taxonomic scale (species-specific within and among reef patterns). At coarser scale (generic pattern across regions), patterns were more uniform (regionally consistent generic dominance on differently exposed reef slopes and at different depths). Neither fine- nor coarse-scale patterns aligned along the sampled 1700 km latitudinal gradient. Thus, a latitudinal gradient that had been described earlier from comparable datasets, separating the Red Sea into three faunistic zones, was no longer apparent. This may indicate subtle changes in species distributions. Coral size, measured as corrected average intercept of corals in transects, had decreased from 1997 to 2009, after having remained constant from 1988 to 1997. Recruitment had remained stable (∼12 juvenile corals per m^2^). Size distributions had not changed significantly but large corals had declined over 20 years. Thus, data from a wide range of sites taken over two decades support claims by others that climate change is indeed beginning to show clear effects on Red Sea reefs.

## Introduction

Climate change is a major threat to the future of the world's coral reefs [1] and reports from the Red Sea suggest that coral growth has slowed [2]. In general, it has been suggested that coral communities may change in future [1] and that many coral species may disappear [3], which may result in more homogenous coral communities [4]. Such scenarios provide testable hypotheses against long-term datasets. The Red Sea's location provides a convenient gradient for studies of latitude-dependent global-scale impacts, such as acidification and ocean heat-content. Also significant local impacts have occurred due to rapid human population growth and urbanization, coastal construction, tourism, reef over-usage [5], [6] and destructive fisheries [7].

**Figure 1:**
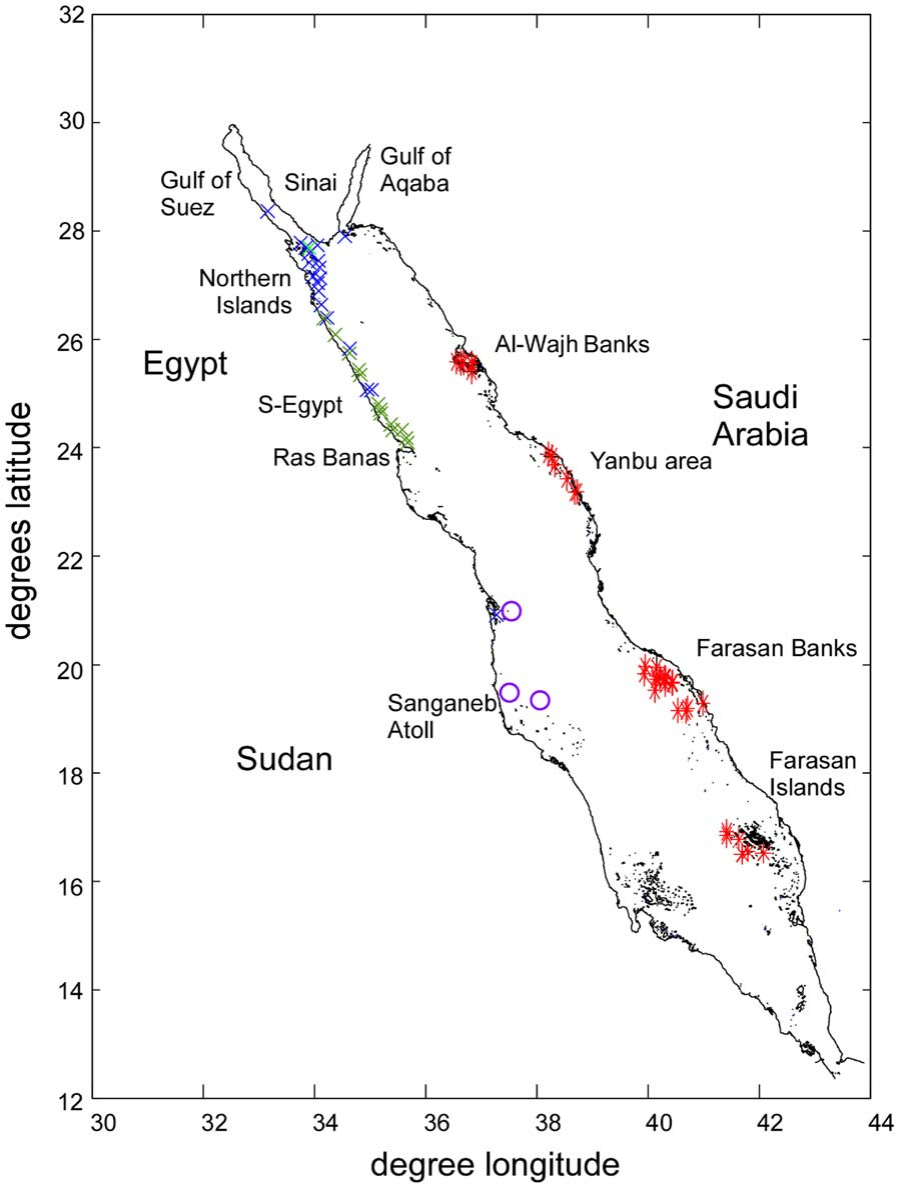
Location of sampling sites in the Red Sea. Red stars = transect surveys 2006, 2008, 2009; blue crosses = transect surveys 1988, 1989, 1997; green crosses = squares of 1997. Sites in Egypt from the Gulf of Suez to Ras Banas from [5], [18], [20]; Sites in Sudan (purple circles) from [13], [17], [26] were used to compare validity of results.

We sampled along a 1700 km gradient from the Gulf of Suez with some of the northernmost reefs in the western Indo-Pacific, to the Farasan Islands of tropical latitude in southern Saudi Arabia (Fig. 1). The Red Sea contains a typical Indo-Pacific fauna with many peripheral endemics [8], [9] and a N-S gradient in species richness and community diversification was described [9]. It is a data rich environment, with reef studies dating to the 18^th^ century and assessment and monitoring work since the 1970s [5], [9], [10], [11], [12], [13], [14], [15], [16], [17], [18], [19], [20], [21], [22] that is useful for comparison.

In the present paper, we evaluate several datasets obtained along the length of the Red Sea by the same researchers over the span of two decades. This rich dataset allows exploration of patterns driven either by spatial or temporal determinants. We examine characteristics of areas sampled at different times, different latitudes to see whether described gradient and patterns [8], [9], [15], [18], [22] remain observable or whether intervening impacts (bleaching events, crown-of-thorns starfish outbreaks, increased coastal construction and pollution, increased fisheries, etc.) caused changes.

## Results

Analyses included 193 transects from a total of 63 sites grouped in four regions (Fig. 1) that were evaluated over a 20-year time span. Absolute species richness recorded in any given transect ranged from 1 to 23 species. Richest transects were found in the Farasan Islands (18–23 spp.) and the Egyptian Northern Islands at Gubal saghir (18 spp.). The naturally most depauperate transects were from the Gulf of Suez, shallow fringing reef crests near Hurghada and in the Farasan Islands (only *Stylophora pistillata*). In the Farasan Islands, Farasan Banks and the Yanbu area, predation by the crown-of-thorns-starfish (COTS, *Acanthaster planci*) had denuded some reefs prior to sampling and only very few, or single, species and/or colonies were encountered (*Diploastrea heliopora* as the most frequently remaining coral). Species dominance of surviving corals reflected the taste of the predator [23] or chance escape, but not ecological differentiation. The Gulf of Suez reefs were unique within the samples and different in community and geological structure [20] and therefore formed a natural outlier in the dataset.

**Figure 2:**
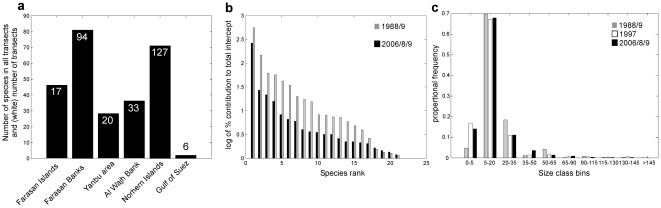
(a) Variation of recorded species richness among sites. (b) Abundance distribution (log) of the 21 most common (>1% of total species occurrences) species encountered in all transects ranked by their relative contribution to total intercept. The species constributing most to total intercept was *Porites lutea* (Table 1). No significant differences (X^2^ = 2.6, df = 15, p>0.25) (c) Size distribution of individual intercepts. No significant differences (X^2^ = 0.14, df = 18, p>0.25).

The Farasan Islands had the richest fauna (Fig. 2). Differences between the exhaustively sampled Farasan Banks and Northern Islands suggest that there indeed exists a decreasing gradient in species richness from the southern into the northern Red Sea. Transects in the present study recorded 139 species, which is more than recorded in the faunistic analysis by [9] (121 spp.), and about half of the 159 spp. reported from Saudi Arabia by [8]. This discrepancy was the result of us (a) lumping closely related species that could not be unequivocally distinguished in the field, (b) use of a taxonomy [9] that recognizes fewer species than [8], [24] in order to maintain compatibility with our 1988 dataset, (c) the limited number of habitats represented in the samples (concentrating on ocean-facing offshore sites, following [9]), and (d) the fact that exhaustive sampling for rarer species was not undertaken (in contrast, a specific goal of [8]). The most frequent and therefore ecologically most important (or most dominant) species as well as the widely distributed rare species were indeed captured by our analyses. Many more rare than common species were found which is shown by only 21 species contributing >1% of total intercept in all areas (Fig. 2). The dominant species were the major zone-formers: *Porites lutea* (dominating leeward reef crests and slopes, but common everywhere), *Stylophora pistillata* (dominating shallow areas, but common everywhere), *P. (Synaraea) rus* (dominant on reef slopes and in coral carpets), *Millepora dichotoma* (dominant in semi- and current-exposed areas everywhere) and *Acropora cytherea* (dominant on wave-exposed reef-slopes) in Saudi Arabia, *A. hemprichii* and *A. valida* in Egypt. Within each sampling area, the composition of locally dominant species varied (table 1).

**Table 1:** Species with the highest intercept values and their relative contribution to overall cover.

Farasan Banks	Yanbu area	Al Wajh Bank	Farasan Islands	Northern Islands	Gulf of Suez	Overall
*Porites lutea* (19%)	*Pocillopra verrucosa* (27%)	*Porites lutea* (14%)	*Porites lutea* (20%)	*Porites lutea* (19%)	*Stylophora pistillata*	*Porites lutea*
*Porites (Synaraea) rus* (9%)	*Porites lutea* (23%)	*Porites columnaris* (12%)	*Acropora clathrata* (14%)	*Porites (Synaraea) rus* (7%)	–	*Stylophora pistillata*
*Acropora pharaonis* (7%)	*Pocillopora damicornis* (6%)	*Acropora forskali* (10%)	*Acropora forskali* (7%)	*Millepora dichotoma* (7%)	–	*Porites (Synaraea) rus*
*Acropora clathrata* (6%)	*Acropora gemmifera* (5%)	*Favia stelligera* (6%)	*Montipora monasteriata* (7%)	*Acropora valida* (6%)	–	*Millepora dichotoma*
*Diploastrea heliopora* (6%)	*Acropora lamarcki* (4%)	*Acropora lamarcki* (6%)	*Acropora cytherea* (4%)	*Acropora cytherea* (6%)	–	*Acropora cytherea*

The sampled sites in the Gulf of Suez were monospecific (not the case in all Gulf of Suez reefs).

**Figure 3:**
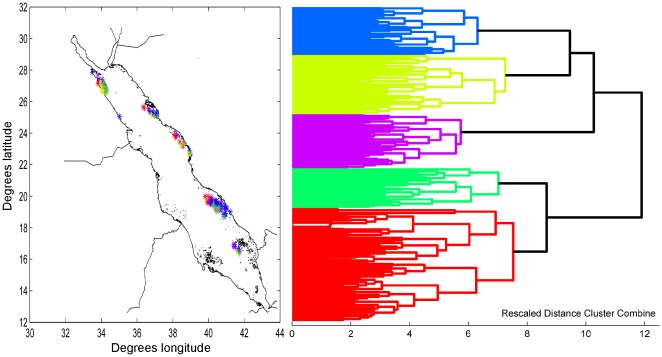
Grouping of transects based on species presence/absence. All clusters include transects from all geographic locations. Euclidian distance with Ward's method of linkage.

Clustering of datasets inclusive of all data from all regions (obtained in 1988/9, 1997, 2006–9 from Egypt and Saudi Arabia) showed consistency at optimal clusters level determined by maximizing the cophenetic index. No separation of samples into unique clusters according to year of sampling occurred, suggesting temporal consistency of data and absence of a primarily temporally-determined pattern (i.e. transit along a successional gradient and/or significant degradation). In all analyses, clusters included transects from all sites (Fig. 3) and no regionally-unique clusters were formed, suggesting absence of a primarily spatially-determined pattern. These results were similar for datasets recording only presence/absence data or intercept values.

Analysis based on presence/absence data showed no separation of any site groups based purely on latitudinal position or year of sampling, suggesting that these were not the main factors determining variability. Use of the same clustering algorithm as in [9] failed to reproduce a similar pattern that differentiated samples from the northern, central, and southern Red Sea. Other clustering algorithms gave better-defined clusters, but these again included transects from all sites and did not arrange orderly along the latitudinal, neither the temporal, gradient. By computing squared Euclidian distance with Ward's method of linkage of log-log transformed species intercept data, twelve clusters were obtained of which one was unique to the Northern Islands, and two to the Farasan Islands. However, all other clusters included transects from all other sites (including the Northern and Farasan Islands) again failing to produce the clear latitudinal differentiation observed by [9]. This suggests that in our dataset, only specific environments but never entire reefs showed unique patterns based on variable species-abundances and presences. Clustering by untransformed proportional intercept of genera (all species summed to genus level), showed no difference among regions but suggested a gross community differentiation into windward *Acropora* slopes and leeward *Porites* slopes and coral carpets, as previously described from the region [9], [13], [16], [17], [18], [19], [20]. A mixed group of transects from semi-exposed *Millepora*-dominated sites and sites of mixed dominance had greater mean depth than the *Acropora* and *Porites* dominated clusters, but differences were not significant.

The pattern of clustering suggested high variability of community pattern on a fine scale (species-specific pattern within individual reefs or among reefs within a region) but absence of a consistent gradient across the entire study area. This means that coral communities in similar setting on nearby reefs could be characterized by a variety of species, but that most of these species-specific community differentiations were not unique to any region. Where unique communities were found, these only occurred in specific environments alongside more widespread community types on nearby reefs. On a coarser, generic level resolution, our dataset suggested more uniformity of pattern and the presence of relatively homogenous differentiation throughout the entire study region. In combination, these findings hint at the potential loss of regionally unique patterns as previously observed by [9].

**Figure 4:**
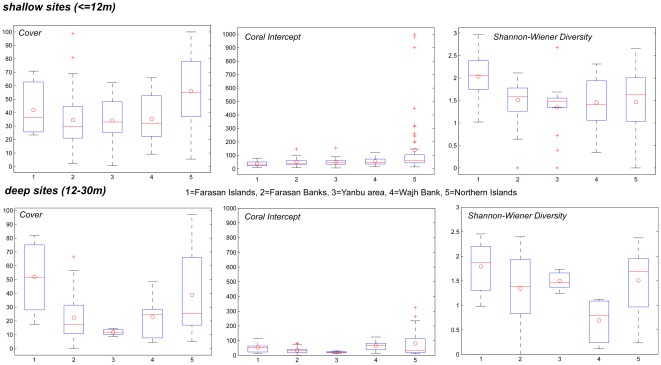
Summary statistics of transects from each survey area (Northern Islands = 1988/9, 1997; Farasan Islands = 2006; Yanbu area and Al Wajh Banks = 2008; Farasan Banks = 2009).

Comparisons of summary statistics between sampling areas and years show absence of statistically significant differences in either coral cover, untransformed coral intercept length as a proxy for coral size distribution, diversity or dominance indices (Fig. 4). There was a tendency for higher coral cover in the 1988/9 dataset than on sites in comparable latitude in 2008/9. Differences between shallow and deep sites were greater than differences among years or among sites. Significant differences in coral cover existed neither between the transect surveys nor in comparison with a dataset of 2×2 m squares from 1997 (average cover 40+/−27% S.D., which falls within the range measured in transects). Thus, neither a strong temporal nor latitudinal effect was evident.

Except in the Farasan Islands, coral cover was mostly higher on shallow reefs. Deep transects in the Egyptian Northern Islands (and data from 2×2 m squares from the Gulf of Suez to Ras Banas) showed high coral cover on dense *P. (Synaraea) rus* and *P. columnaris* coral carpets (biostromes [19], [20]). Similar habitats in the Saudi Red Sea had succumbed to bleaching and/or COTS outbreaks by 2008/9. Coral intercepts were highest in 1988/9 in the Egyptian Northern Islands and many particularly large colonies show as outlier values (\. 4). Absence of comparable values in the Saudi data from comparable habitats with comparable communities is considered an indication for loss of many large colonies, potentially due to the 1998 bleaching event. Average coral size calculated from corrected intercepts [25] suggested that it had declined (1988/9: 18.4 cm, 2008/9: 16.9 cm). The main reason for declining average chord-length of intercepted corals in 2008/9 was a noticeable reduction of large *Porites* and *Acropora*. Size distributions of intercepts did not change significantly from 1988–1997–2008 (Chi-square tests, p>0.05), since corals in the largest size-classes had always been rare (Fig. 2).

Average recruit density in the Farasan Banks in 2009 was 3.3+/−1.7 S.D. juvenile corals (<5 cm diameter) per sampling unit, suggesting 13 juvenile corals per m^2^. Absence of change in coral size distributions among surveys (Fig. 2b) may also be considered evidence for absence of changes in recruitment.

## Discussion

Results from three reef assessments in the Red Sea over two decades suggest temporally consistent and scale-dependent community variability (higher at geographically and taxonomically fine scales) with overall little ordering along a latitudinal gradient as well as a decline in average coral size. While the former suggests potentially system-wide changes in comparison to previous findings [9], the latter is consistent with other studies suggesting impacts by past bleaching [26] and declined coral growth rates [2].

Our datasets were internally consistent, since all surveys were executed and evaluated by the same person using the same method (10-m-long line intercept transects). The adequacy of transect length had been tested [5], [18] and was maintained throughout. But methods deviated from important comparative studies [8], [9], that used a “roving method”, estimating coral cover by eye while attempting to record species presence/absence to finest detail. However, the present study increased taxonomic resolution (139 against 121 species recorded in [9]) which therefore cannot be the cause for failure to detect the same latitudinal pattern with the same methods.

While [9], [15], [22] showed an ordering of community pattern from the Gulf of Suez to the central and southern Red Sea, absence of statistical groupings in the presently evaluated transects suggested absence of such an ordered gradient of communities throughout the length of the Red Sea. Even with the exact same analysis as in previous studies (hierarchical clustering using group average method based on Jaccard distance measure [9]), only many small clusters, unordered along the N-S gradient, could be obtained. Using a different method (hierarchical clustering using Ward's method based on squared Euclidian distance), groupings could be generated that at best separated extremes as subclusters of bigger groupings again containing transects from all regions. Such extremes were shallow habitats in the Gulf of Suez and Farasan Islands (dominated exclusively by *S. pistillata*) and some deep Farasan Island transects (dominated by *A. clathrata* and *A. downingi* that do not occur in the N). Thus, while the present dataset found evidence for regional variability, a clear picture of absence of an ordered latitudinal gradient of community pattern emerged. These results are compatible with patterns observed along a similar spatial gradient in the Great Barrier Reef [27]. Comparable to [22], the present dataset still showed some exclusive species occurrences but such locally unique characteristics were drowned out by regional commonalities. Therefore, the lack of observable differentiation of community pattern across the latitudinal gradient must probably be considered a new phenomenon and may be indicative of a decline of previously demonstrated [9] taxonomic distinctness among regions.

Like elsewhere, Red Sea coral reefs have been subjected to serious disturbances over the past few decades. The 1998 bleaching event affected reefs in Saudi Arabia [26] and in Sudan [28], but apparently less so in Egypt and the northern Gulfs [29], [30]. The Northern Islands region has been impacted by anthropogenic degradation [5], [6], dynamite fishing [7], and several COTS outbreaks [31; pers.obs.]. Also Saudi reefs were impacted by ongoing COTS outbreaks during the present surveys (Al Wajh Banks, Farasan Banks, Farasan Islands) and had clearly suffered from past bleaching (Farasan Islands, pers. obs.). Many almost completely denuded reefs were encountered. Some of the rarer species recorded by [9], [22] were no longer found in our later surveys (e.g. *P. nigrescens*, *A. grandis*). This may suggest that some of the rarer coral species, some of which were responsible for the latitudinal gradient observed by [9], have declined to a point where they no longer contribute in a quantitatively relevant way to community composition. Also potential northward expansion of species previously only recorded in the southern Red Sea (e.g. *A. muricata*, *A. nobilis*, *Montipora stellata* recorded by [9] only from the southern Red Sea, but by [8] and us as far N as the Al Wajh Banks) may be now obscuring previously observed latitudinal patterns.

The most common, zone-forming genera and species showed consistency throughout the sampling area. On upper reef slopes (<12 m), the typical dominant corals were *Acropora* on the more exposed sides, *Porites* in lagoons, more sheltered areas and in many coral carpets, and *Millepora* at current-exposed sites. This is in accordance with observations in the literature [8], [9], [10], [13], [15], [16], [18], [19], [20], [22], [32], [33]. Locally, varieties to this pattern exist but a generalized, region-wide pattern in generic dominance persists nonetheless.

Previous studies in the Gulf of Aqaba [12], [30], [34] and Sudan [21], [35] showed higher coral population turnover in the N and more community constancy in the S (Sudan; [13], [21]). Thus, average colony size in Saudi Arabia should have been larger than in Egypt's Northern Islands, not smaller as recorded by us (Fig. 4). This decline in coral size can likely be attributed to major disturbances within the past two decades. Many large corals were killed in bleaching events and COTS outbreaks, reducing average coral size in the central and southern Red Sea. In particular, upper sheltered reef slopes dominated by *P. lutea* and medium deep *P. (Synaraea) rus* and *P. columnaris* carpets that contained exceptionally large and healthy colonies in the 1980s and early 1990s [18], [20], [33] were found with many dead corals in the later surveys in Saudi Arabia. Disturbances were still continuing during the surveys but where measured, recruitment levels were high. Thus, the smaller average coral size in the later surveys clearly shows the effects of stresses but the unchanged high recruitment rates may also be indicative of community resilience [36].

In conclusion, our study points to potentially increasing community homogenization and decline in average size of coral colonies throughout the Red Sea. These are phenomena that have also been observed in other peripheral seas. Different to the Caribbean, where community homogenization results from loss of dominant species and replacement by novel, but region-wide occurring communities [4], we propose that the mechanism in the Red Sea is one of increasing loss of diversity, particularly of rarer species, and potential distributional (northwards) shifts. The decline in average coral size is ascribed to increased COTS predation, bleaching mortality, and coral diseases. Unchanged recruitment levels, however, suggest that while coral communities may have been subtly altered, they presently still remain resilient to wholesale decline.

## Methods

Coral communities were sampled on Egyptian and Saudi Arabian offshore reefs between the Gulf of Suez and the Farasan Islands, a latitudinal gradient of ∼1700 km, by means of 10m-long line-intercept transects [37], 2×2 m squares and 10m-long phototransects. The first dataset was taken on reefs between the southern Gulf of Suez and the Hurghada area, centered on the Northern Islands, in Egypt in January 1988 and 1989 [5], [18]. The second set was taken in the same area in 1997 [20]. The third dataset was taken in Saudi Arabia in 2006 (Farasan Islands), 2008 (Yanbu area, Al Wajh Banks) and 2009 (Farasan Banks, [36]) (Fig. 1). During all surveys, transects were placed in a stratified random pattern at depths centered on <5 m, 10 m, 20 m, 25 m, allowing two meters up or down adjustment to accommodate reef morphology and other constraints during diving. In 1988/9 and 1997, regular line-intercept transects [37] were recorded. Between 2006 and 2009, overlapping photographs along a measuring tape creating 0.5×10 m photo-corridors were taken. Images were merged and gridded to unit pixel-size (1 pixel = 1 mm^2^). A line down the center was then taken as the transect-line equivalent and intercepts of all encountered corals were measured to the nearest mm. All transects were recorded and evaluated by the same author (BR), eliminating observer-specific identification or measurement variability. Coral cover information from sites between Hurghada and Ras Banas obtained by visual estimation of cover in 2×2 m sampling squares in 1997 were also used for comparison. Adequacy of transect length and quadrate size had been tested previously [5], [18].

For comparison of community pattern based on relative coral cover per species/genus, all individual coral intercepts were pooled per taxonomic unit. To evaluate colony sizes, a dataset of intercept measurements for every single encountered coral was produced. Coral intercept is not a simple measure of coral size (diameter), since neither the position of intercept on the coral, nor the distribution function of either the intercepts or of the underlying coral diameters are known. From the datasets of individual coral intercepts, considering them chord-lengths intercepting circles in the plane, the relationship developed by [25]:
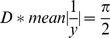
(1)was used to calculate mean diameters of corals (*D*, approximated as non-overlapping circles of an unknown distribution in the plane) using the harmonic mean of the chord lengths *y* (i.e. coral intercepts on transects). Also cumulative intercepts per species can be a useful measure. If intercepts were to markedly decrease among surveys, this, together with calculation of *D* (eq. 1) could be counted as indication for decline of large corals. Both approaches were used to evaluate stationarity or change of average coral size. Size distributions of intercepts were corrected for bias following [38]:
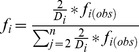
(2)Where 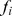 is corrected frequency and 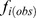 is the frequency observed in the transects.

Species names were harmonized between surveys to account for different taxonomic interpretations [9], [24]. For community analysis, transects were subjected to cluster analysis, the most commonly used method in comparable studies [8], [9], [15], [18], [20]. Agglomerative, hierarchical cluster-analysis with Ward's method of linkage and the Euclidian distance metric was preferentially used, or, for reasons of comparability, Jaccard's index for binary (presence/absence) data and group average method of linkage [9]. As indication of clustering quality the cophenetic correlation index, relating to correlation between the original similarities and similarities of samples, was calculated [39]. The closer to unity, the better the dendrogram reflects original among-sample distances. Clusters were chosen to obtain the highest cophenetic index. To characterize individual transects, total cover as the summed intercepts in each transect, average intercept of corals per transect, and Shannon-Weaver diversity were calculated.

Coral recruitment was evaluated from the unbiased [37] counts of corals <5 cm in transects and also in 2009 in the Farasan Banks by counting all corals <5 cm diameter within 25×25 cm squares (N = 580), five of which were placed haphazardly within the area of a phototransect.
